# St. Paul's Cathedral, June 13, 1919

**Published:** 1919-06-21

**Authors:** 


### THE ROYAL NAVY.

### ST. PAUL'S CATHEDRAL, JUNE 13, 1919.

One of the most beautiful memorial services was that held in memory of officers and men of the Royal Navy who have given their lives in the service of their King and Country during the war, 1914-18. Thoroughness, the mainspring of all the Navy does, was brought home to everyone present when they entered St. Paul's at the commencement of the service.. While the congregation was assembling a programme of music was played by the band of the Royal Marine Light Infantry (Chatham Division), under the direction of Lieutenant J. C. J. Hoby, R.M.L.I., Mus.Doc. Oxon. It comprised Overture in C "In Memoriam," Sullivan; Largo in G, Handel; Allegro con Grazia (Symphonie Pathétique), Tchaikowsky; Meditation, Bizet; and Marche Funèbre, Beethoven. These formed a moving and appropriate prelude to one of the most impressive services ever held in St. Paul's Cathedral. We could wish the Cathedral authorities would select for solemn occasions like that of the 13th inst. readers who have a commanding voice and distinct utterance. Portions of the service were difficult to follow from indistinctness, which was felt keenly by all who entered heart and soul into the service. The music was of the very best—could not have been better. The anthem by Stanford was taken from Revelation xiv., v. 13, "I heard a voice from heaven, saying unto me, Write, from henceforth blessed are the dead which die in the Lord; even so saith the Spirit; for they rest from their labours, and their works do follow them." The Lesson, which was the Navy's own choice, was taken from Revelation xx. The chapter concludes:

And I saw a great white throne, and Him that sat on it, from Whose face the earth and the heaven fled away; and there was found no place for them.

And I saw the dead, small and great, stand before God; and the books were opened: and another book was opened, which is the book of life: and the dead were judged out of those things which were written in the books, according to their works.

And the sea gave up the dead which were in it; and death and hell delivered up the dead which were in them: and they were judged every man according to their works.

And death and hell were cast into the lake of fire. This is the second death.

And whosoever was not found written in the book of life was cast into the lake of fire.

The previous day, June 12, had been a wonderful day in the City of London, when Admiral Sir David Beatty and Field-Marshal Sir Douglas Haig went to the Guildhall to receive the freedom of the City of London. That ceremony was full of impressive and thrilling moments, and who will ever forget Sir David Beatty's concluding words to his speech. "There was never a time in its history when the Navy has been more of one mind than at present. One of the greatest traditions of the Service is loyalty; loyalty to King, loyalty to Country, and loyalty within itself. I trust that this great tradition will never be lost sight of," and then raising his head, in ringing tones, "**I trust it will be always remembered that our Empire lives by the sea, and that the Royal Navy is loyal steadfast and true.**"

The present writer remembers once, when our beloved King, the late King Edward, was discussing the Navy, he asked if it was not the fact that the training of the British Navy was the finest and best training a man could have? He added, "I have reason to realise this great fact," and it is certain that our present sailor King knows it to be true, and has demonstrated its force and value since he ascended the throne of England.

### The Navy's Share in Victory.

The atmosphere of St. Paul's on June 13 was charged with the sense of the splendid service rendered by our sailors of all ranks. The conduct of officers and men in action, which has characterised all the armed forces of the Empire, and covered them with glory, in modern battle at sea as on land, has exacted heavy toll. How severe a toll is shown by the fact that the losses on both sides at Jutland far exceeded those of any other sea battle in history. We landsmen must ever have in our recollection the fact that for men of the fighting Services one of the hardest things to bear was monotony. It was the lot of the Fleet to carry out endless patrols, sweeps, reconnaissances, frequently without sighting anything except a periscope, yet day by day the sailors read of their fathers, brothers, and friends flocking to the Colours for the defence of their Country, engaged in glorious achievement, winning laurels, and gladly making the supreme sacrifice in the face of an enemy whom they themselves seldom met. So Admiral Beatty testifies, and, despite these heartburnings, the Fleet had to settle down to its plain duty of holding the command of the sea. True, this is no new phase of naval warfare. After the Battle of the Nile the Fleet had to wait seven years for its next great action off Trafalgar.

### The Merchant Sailor.

The story of the naval war has been embellished by the wonderful record of the Mercantile Marine. Much has been told and much more remains to be told of their indomitable courage and loyalty, without which the war would not have been won. None are more proud of them than are their comrades in the Royal Navy. The close association which has been established between the two Services during the war must continue, so that the sailors may remain the one great Sea Service of the Empire. Every landsman, while he rejoices and honours our sailors, has an opportunity at the present time, when in a position to do so, to make a point of enlisting near his

**The Royal Navy**—(*continued*).

person a British sailor, whose training has given him invaluable qualities of resource, loyalty, discipline, experience in handling men, and an adaptability to circumstances, making him, as Admiral Beatty has testified, peculiarly fitted for many of the positions which have to be filled in civil life.

THE MEMORIAL OF THE DEAD.

Yes, we must remember our seamen of all ranks, honour and love them, and teach our children to reverence and trust them too, remembering all that our sailors have accomplished for the Empire and the world during the war, with hearts touched, and desire excited to honour and sustain our Navies and all connected with them.

We were awakened to the realities of the moment by a tender voice which proceeded, "Let us remember with thanksgiving and all honour before God and men, all ranks and ratings of the Royal Navy, Royal Marines, Royal Naval Reserve, Royal Naval Volunteer Reserve, Royal Naval Air Service, Royal Naval Division, Minesweeping Service, Coast Watchers, Queen Alexandra's Royal Naval Nursing Service, Women's Royal Naval Service, Mercantile Marine, and Sea Scouts, who gave their lives for their King and Country in the great war."

Silence was kept for a space, then the souls of the faithful were commended to the keeping of Almighty God. Then came this beautiful prayer for all engaged on the sea.

"O Eternal Lord God, Who alone spreadest out the heavens, and rulest the raging of the sea; Who hast compassed the waters with bounds until day and night come to an end; be pleased to receive into Thy Almighty and most gracious protection the persons of Thy servants the officers and men of our Navy, and the Fleet in which they serve; preserve them from the dangers of the sea and from the violence of the enemy, that they may be a safeguard unto our most gracious Sovereign Lord, King George, and his dominions, and a security for such as pass on the seas upon their lawful occasions: that the inhabitants of our Empire may in peace and quietness serve Thee, our God; and that those who are on the deep may return in safety to enjoy the blessings of the land, with the fruits of their labours, and with a thankful remembrance of Thy mercies to praise and glorify Thy Holy Name: through Jesus Christ our Lord. Amen."

Following this came the prayer for those whom war had made desolate and broken-hearted. Finally the audience prayed for themselves that they might be worthy of those who have given their lives for their King and Country. The service concluded with the hymn, sung with heart and voice by all present, "For all the Saints who from their labours rest" from Hymns Ancient and Modern. Then followed the Benediction, the Dead March (Chopin) magnificently rendered, Last Post, Reveille, "God Save the King," and the Thanksgiving March.

